# “Stop and just breathe for a minute”: perspectives of children on the Autism Spectrum and their caregivers on a Mindfulness Group

**DOI:** 10.1007/s10803-022-05542-x

**Published:** 2022-06-30

**Authors:** Megan K. Hatfield, Emma Ashcroft, Siobhan Maguire, Lauren Kershaw, Marina Ciccarelli

**Affiliations:** Curtin School of Allied Health, Curtin University, GPO Box U1987, 6845 Perth, Western Australia Australia

**Keywords:** Autism, Anxiety, Mindfulness, Children, Group, Occupational therapy

## Abstract

Children on the autism spectrum often experience anxiety. Mindfulness is growing in popularity as a method to support children with anxiety; however, there is limited evidence on mindfulness for children on the autism spectrum. This study investigated the feasibility of a 10-week group-based mindfulness intervention for 14 children on the autism spectrum. A one-group pre-post design determined outcomes of anxiety (caregiver and child report), mindful attention, and wellbeing (child report). Interviews explored children’s and caregivers’ perceptions. There were no significant differences in outcomes post-intervention. Five themes were identified from interviews: (1) Children felt calmer; (2) Parents observed differences in anxiety; (3) Breathing and yoga helped; (4) Parents wanted more; and (5) Challenges and benefits of group intervention.

## Introduction

Children on the autism spectrum share some common characteristics including difficulties with social interactions, combined with limited and repetitive interests and behaviours (American Psychiatric Association, 2014). Children on the autism spectrum experience higher levels of anxiety compared to neurotypical children (van Steensel & Heeman, 2017; White et al., 2009). Parents of children on the autism spectrum report that anxiety has a negative effect on their child and the family (Beato et al., 2018), including being unable to verbally express their worries and concerns that can result in avoidance, arousal, sensory seeking behaviours, and repetitive behaviours (Ozsivadjian et al., 2012; Simpson et al., 2020). Parents have reported that these anxiety-related behaviours limited their child’s abilities to study or make friends, isolating them, and stopping them from experiencing new things (Beato et al., 2018).

Mindfulness developed from the foundations of Buddhist meditation and has been used as a anxiety reduction strategy in various settings (Kabat-Zinn, 2011). Characteristic elements of mindfulness include the awareness of breathing, bodily sensations, actions, thoughts and feelings, and shifting attention (Hwang & Kearney, 2013). In mindfulness interventions, participants are trained to focus on the present while maintaining a non-judgemental and open-minded attitude to become aware of any negative emotions or feelings they are experiencing (Ridderinkhof et al., 2018; Segal et al., 2012). Mindfulness for children has been associated with improvements in executive function, with increased improvements noted for children with lower executive function at the start of the intervention (Flook et al., 2010). Mindfulness interventions have the potential to support improved communication and social skills in children on the autism spectrum because it supports being in the moment with another person and increased awareness of one’s own and other’s emotional states (Ridderinkhof et al., 2018; Segal et al., 2012).

There is a growing body of evidence that supports the use of mindfulness interventions in reducing anxiety and depression in adults on the autism spectrum (Kiep et al., 2015; Sizoo & Kuiper, 2017; Spek et al., 2013; White et al., 2018). However, less research has explored the feasibility and effectiveness of these strategies for children on the autism spectrum. There are two known mindfulness programs designed specifically for children on the autism spectrum that have published evidence. The MYmind program for children on the autism spectrum and their parents has been found to support social communication, behavioural and emotional functioning (Ridderinkhof et al., 2018; Ridderinkhof et al., 2020), increased quality of life, and decreased rumination (de Bruin et al., 2015). Another intervention focused on building parents’ capacities to teach mindfulness techniques to their children on the autism spectrum has been found to reduce the child’s anxiety and maladaptive thoughts (Hwang et al., 2015). A meta-analysis of mindfulness interventions for people on the autism spectrum found that including one or both parents in the mindfulness intervention for children may improve the child’s subjective well-being at follow up (Hartley et al., 2019). However, prior research has not examined the effect of mindfulness without direct parental involvement as an intervention for children on the autism spectrum. In addition, no studies have explored the perspectives of the children and their parents using qualitative methodologies. To address the gap in the research evidence about the potential benefits of mindfulness with children on the autism spectrum, a group-based mindfulness intervention, Mindful Movers, was developed, trialled, and evaluated using quantitative and qualitative methodologies. The aim of this study was to investigate the feasibility of a group-based mindfulness intervention for children on the autism spectrum.

## Methods

### Study design

This exploratory study used a one-group pre-post design to determine differences in outcomes before and after participating in the Mindful Movers program, and a phenomenological qualitative approach was used to gain a deeper understanding of the experiences and perceptions of the children on the autism spectrum who participated in the mindfulness program, and their caregivers. Recent evidence highlights the importance of using a mixed-methods design in mindfulness research to explore the application of mindfulness to new populations and to capture the complexity of the experience (Huynh et al., 2019). Our study utilised a convergent mixed-methods design, where the qualitative and quantitative data were collected at similar time points and the findings merged at the discussion (Plano Clark & Ivankova, 2016).

### Participants

Children aged 10 to 14 years with an autism diagnosis (according to the Diagnostic and Statistical Manual of Mental Disorders ([DSM-5]; American Psychiatric Association, 2014) and their parent or guardian were invited to participate in the study. The children were eligible for inclusion in the study if the family had self-identified a therapy goal related to the management of anxiety or self-regulation as part of the goal-setting process for their disability service provider; were proficient in conversational English (spoken or via assistive communication devices); and able to participate in group-based sessions.

All participants were recruited from a not-for-profit disability service provider located in [location removed for blinding during review], who delivered the Mindful Movers program. The disability service provider operated outside of schools but worked in collaboration with schools and any other community services. The disability service provider forwarded the recruitment flyers for the study to caregivers of eligible children who were enrolled in the Mindful Movers program. Children could participate in the Mindful Movers program without participating in the study. Parents/guardians provided written informed consent for themselves and their children to participate in the study, and the children provided their written assent. Fourteen children participated in the Mindful Movers program, and all participants took part in the same group. Of these, 10 of the children and their caregivers completed the quantitative outcomes measures. Nine children and 10 caregivers consented to be interviewed for this study. Where necessary, participants’ names were replaced with pseudonyms when reporting the results to maintain confidentiality. The [institutional ethics committee] approved the qualitative and quantitative components of this study (approval# [numbers removed]; respectively).

### The Mindful Movers Program

The Mindful Movers program was developed to help children on the autism spectrum understand the concept of mindfulness and teach them strategies to support emotional regulation skills. The group-based intervention was delivered by two occupational therapists. One occupational therapist was also a qualified yoga and mindfulness teacher. The other was qualified in teaching yoga to children. Both were involved in the development of the Mindful Movers Program and had run the program previously. The program was delivered at the community-based premises of a not-for-profit disability service provider once each week for 10-weeks; each session was 75 min in duration. Each session began with a welcome and emotion check using an app. Session activities included mindful movement (e.g. yoga and stretching), breathing (e.g. the 4-7-8 technique), and meditation guided by the therapists (see Table 1 for more details). These activities were included to improve the children’s awareness of their bodily sensations and thought processes. Activities involved participants engaging in cognitive activities that supported their understanding of being in the present moment and being able to view their thoughts with neutrality and without judgement. One of the activities in the sessions was ‘Zen questions’, where participants engaged in asking themselves questions to be more aware of the present moment and the content of their current thoughts. In another activity, participants engaged in meditation where the objective was to observe thoughts and feelings without judgment. The Mindful Movers program included activities for the children to complete outside of the group, that supported their integration of concepts taught into their everyday experiences. In the subsequent week’s session, therapists led discussions around if/how participants engaged in strategies outside of the group and provided support to enhance subsequent use of strategies between sessions. Parents were not directly involved in sessions with the children. In the final 15 min of the intervention sessions, one of the therapists facilitated a group discussion with the parents/guardians in another room away from the children about the concepts learnt in the session, and how to support their children to apply these strategies outside of the group.

**Table 1:** Activities completed in the Mindful Movers group

Movement		Mindfulness		Breathing		Meditation		
• Sun salutation • Group circle holds • Martial arts • Yoga poses		• Mindful or mind full awareness • Mindful colouring • Mindful dot on hand • Grounding, sense countdown • Zen questions • Glitter jar • Mindful eating		• Blowing games – e.g., blowing pom-poms • Flower/leaf visual • 4-7-8 technique • Altering heart rates with metronome • Breathing techniques: Tummy, back-to-back, elephant, take 5, Bumble be-bee, and balloon breathing.		• DIY stress ball • Butchers’ paper anxiety discussion • Body scan • Walking meditation • Progressive muscle meditation • Dancing warriors • Listening bell • Slow hands meditation		

### Outcome Measures

Pre-test outcomes were completed one week before the Mindful Movers program commenced (T1); post-test measures were taken one week after program completion (T2); and follow-up was at two months after program completion (T3). Participants completed three outcome measures at all three time points, as described below. The outcome measures were distributed and scored by an independent researcher who was not involved in the delivery of the program. All outcome measures were self-report. Self-reported assessments completed by children and adolescents on the autism spectrum are considered reliable and valid data (Shipman et al., 2011). Interviews were conducted with parents and children at the participants’ place of choice within one month of the final Mindful Movers program session to gain insight into their experiences and perspectives of the group.

### Mindful Attention Awareness Scale-Adolescents (MAAS-A)

The Mindful Attention Awareness Scale-Adolescents (MAAS-A) was used to determine the child’s level of mindfulness – i.e., awareness of their feelings, experiences of anxiety, and challenging behaviours (Brown et al., 2011; de Bruin et al., 2011). The MAAS-A is a valid and reliable measure of adolescents’ mindfulness traits and has demonstrated strong internal consistency (Cronbach’s α = 0.81) with this population (de Bruin et al., 2015; de Bruin et al., 2011).

### Anxiety Scale for Children – Autism Spectrum Disorder (ASC-ASD)

The Anxiety Scale for Children – Autism Spectrum Disorder (ASC-ASD) is a measure that assesses perceived changes in an adolescent’s anxiety symptoms from their point of view (Rodgers et al., 2016). This tool has demonstrated strong internal consistency and test-retest reliability, and moderate validity (Rodgers et al., 2016). Children in our study completed the ASC-ASD and their parents completed the Anxiety Scale for Children – Autism Spectrum Disorder – Parent version (ASC-ASD-P) to report perceived changes in the child’s level of anxiety.

### World Health Organization- 5 Well-Being Index (WHO-5)

The World Health Organization-5 Well-Being Index (WHO-5; DeWit et al., 2007; Topp et al., 2015) is suitable for use with children aged 9 years and older. It provides a measure of an adolescent’s overall mental health and wellbeing. The measure includes five statements - (i) I have felt cheerful in good spirits; (ii) I have felt calm and relaxed; (iii) I have felt active and vigorous; (iv) I woke up feeling fresh and rested; and (v) My daily life has been filled with things that interest me. Participants respond by indicating how much of the time in the past two weeks they had felt like the descriptor in each statement, with 0 = ‘At no time’ and 5 = ‘All of the time’. The total raw score ranges from 0 to 25, with 0 representing worst possible quality of life and 25 representing best possible quality of life. The WHO-5 demonstrated strong, internal consistency (Cronbach’s α = 0.83) in a previous study of mindfulness meditation among adolescents with anxiety (de Bruin et al., 2015).

### Semi-structured interviews

Interviews with the children were between 14 and 28 min long and the parents/guardians’ interviews were between 16 and 34 min. Interviews were completed by an external researcher who was not involved in the administration of the group in any way. The semi-structured interviews focused on participants’ perceived experiences of the mindfulness group program. Interviews with each child started with a “chatter box” origami game to engage the child and build rapport with the interviewer. Visual aids were used during the children’s interviews, including pictures of what the children had done in the mindfulness group, examples of things they had made, and visual prompts for the techniques that had been included in the group. Children and parents were asked if they used the mindfulness techniques outside of the group. See Appendices 1 and 2; respectively for the interview schedules used with the parents and children.

### Data Analysis

Differences in scores on the scale measures across the three time points (T1-T2 and T1-T3) were analysed with a random-effects mixed regression model in SPSS v.25 (IBM Corp, 2017). A repeated measures test was used in preference to repeated measures analysis of variance because it considered missing values and was appropriate for the small sample in the study (Bickel, 2007). Changes over time in responses to individual items were calculated using Hedges G. As a broad and very general rule of thumb, Cohen (1988) suggested that an effect size of 0.2 represented a small effect; 0.5 a medium effect; and 0.8 a large effect, but these interpretations were used with caution in this study (Durlak, 2009). A p-value < 0.05 was used to indicate a significant difference in scale outcomes across time periods.

Interviews were audio-recorded and transcribed verbatim by one of the researchers and a professional transcription service. Two child participants and one parent participant did not want to be audio-recorded and so detailed field notes that included verbatim quotes were taken during these interviews. Transcripts were analysed using Clarke and Braun’s (2016) six-stage method for thematic analysis: (i) becoming familiar with the text, (ii) coding, (iii) searching for themes, (iv) reviewing themes, (v) defining and naming themes, and (vi) final analysis and write up. The preliminary codes and themes were discussed by two researchers, and further refined by the research team. Consensus was reached by the research team, with any disagreements regarding themes resolved through discussion. Credibility was achieved through triangulation of data from multiple sources (i.e., children and parents/guardians, separate interviews; and collection of quantitative and qualitative data). An audit trail of the thematic analysis was documented using NVivo (Given, 2008; Liamputtong, 2013).

## Results

### Participant demographics

Ten of the 14 children on the autism spectrum who participated in the mindfulness group (females = 3; mean age = 11 years 10 months; SD = 17 months) and their parents completed the scale outcome measures in this study. Nine children and 10 parents/guardians were interviewed (an interview was not able to be scheduled at a suitable time with Child 10). The demographic information of the participants is presented in Table 2. All participants communicated verbally, none utilised alternative communication methods at the time of the group. The greater number of male participants reflects the higher incidence of males diagnosed as being on the autism spectrum within the Australian population (Australian Bureau of Statistics, 2018).

**Table 2:** Participant demographics

Child participants				Parent/guardian participants	
ID	Age (years)	Sessions attended	Biological Sex	ID	Relationship to child
C1	14	9/10	Male	P1	Mother
C2	13	6/10	Male	P2	Mother
C3	13	7/10	Female	P3	Father
C4	13	9/10	Female	P4	Mother
C5	10	7/10	Female	P5	Mother
*C6	10	8/10	Male	P6	Father
C7	11	8/10	Male	P7	Grandparents
*C8	11	6/10	Male	P8	Mother
C9	11	9/10	Male	P9	Mother
†C10	10	6/10	Male	P10	Father

* No consent to record interview; †Interview with child unable to be scheduled at suitable time

### Quantitative Results

#### Child and parent ratings of child anxiety

A score of > 20 on the ASC-ASD and the ASC-ASD-P may indicate the presence of significant anxiety (Rodgers et al., 2016). The mean total scores on the child and parent measures were > 20 at all three data collection points in the study (see Table 3), which indicated the children rated themselves as experiencing anxiety at these times, as did their parents. The children’s ASC-ASD scores did not show any significant changes from baseline to after the program was completed, and the average score remained > 20. Parents’ scores on the ASC-ASD-P of their child’s level of anxiety also did not indicate any change from baseline to follow-up (see Table 3).

**Table 3:** Scores of the child (n = 10) and parent (n = 10) measures at baseline (T1), post-test (T2), and 2-month follow-up (T3)

		Mean [95% CI]		Raw difference (T1- T2)	*P* (T1-T2)	Effect size	Raw difference (T1-T3)	*P* (T1-T3)	Effect size
Outcomes	T1	T2	T3		p	G		p	G
Anxiety Scale for Children – Autism Spectrum Disorder (ASC-ASD)	25[16, 34]	21 [11, 31]	21 [14, 28]	-4	0.324	− 0.35	-4	0.435	− 0.27
Anxiety Scale for Children – Autism Spectrum Disorder – Parent version (ASC-ASD-P)	26 [20, 32]	27 [19, 35]	29 [20, 38]	1	0.898	0.45	-3	0.702	0.13
Mindful Attention Awareness Scale-Adolescents (MAAS-A)	55 [48, 62]	62 [50, 74]	59 [49, 69]	7	0.398	0.30	4	0.581	0.19
World Health Organization-5 Well-Being Index (WHO-5)	15 [12, 18]	16 [13, 19]	15 [11, 19]	1	0.458	0.26	0	0.954	-0.02

Note. G = Hedges G, *p < 0.05

*ASC-ASD & ASC-ASD-P: A score > 20 may indicate the presence of significant anxiety (Rodgers et al., 2015). MAAS-A: The higher the score, the greater the level of dispositional mindfulness (Brown & Ryan, 2019). WHO-5: A score < 13 suggests poor health and wellbeing, and can be used as an indicator for testing depression under ICD-10 (WHO Five Well-Being Index, 1998)*

### Mindfulness

The children’s mean MAAS-A scores did not show evidence of change at follow-up relative to pretest, as shown in Table 3. One item demonstrated a significant change between pre-and post-test that was maintained at the follow-up (p < 0.05); Item 4 ‘I tend to walk quickly to get where I am going, without paying attention to what I experience along the way’. Another item demonstrated a significant difference between pre-test and post-test (p < 0.001); Item 1 on the MAAS-A - ‘I could be experiencing an emotion but not be conscious of it until later’. This difference was not sustained at follow-up (p = 0.555). This suggests that the program supported participants to be more conscious of emotions in the moment, but that this ability was not maintained once the program ceased.

### Mental health and well-being

Children’s WHO-5 total scores were consistent at pre-test (T1) and follow-up (T3). The children’s mean total score was > 13 at all three time points (see Table 3), indicating they were unlikely to experience depressive thoughts or feelings (DeWit et al., 2007; Topp et al., 2015).

### Qualitative Results

Thematic analysis of the interview data resulted in five themes: (1) Children felt calmer; (2) Parents observed differences in anxiety; (3) Breathing and yoga helped; (4) Parents wanted more; and (5) Challenges and benefits of group intervention. These themes represent the reported experiences of the children who participated in the group and the perceptions of the parents. Participant experiences and perceptions of Mindful Movers were mostly shared with some differences explored in the descriptions below.

### Children felt calmer

Most children reported that they experienced a positive change in their affect after participating in the mindfulness group intervention. When asked how they felt before attending the group, children reported they felt “sad a lot, I got angry a lot” (C7) and that they had “too many thoughts” (C4). Multiple children reported that after attending the group they felt “calmer” (C3, C5, C7, and C8); “happier” (C7); “relaxed” (C3); “a lot better” (C1 and C9); and “more clear” (C8). One child described an improvement in their frustration tolerance, saying “I don’t get annoyed as easily” (C2). Other perceived benefits of attending the mindfulness group included improvements in social skills and self-awareness; one child reported, “I can connect to people more easily … I understand myself better. And I can understand other people better as well” (C2). This participant also reflected that it changed his self-image; “I don’t get as mad as often and I can feel better about myself because of that” (C2). One child felt the strategies he had learned in the mindfulness intervention would be helpful for others, saying that he “might share it with my friends who are stressed at school” (C8).

A few children reported that they did not notice any difference in their anxiety levels after participating in the group; that they still felt just as anxious. One child reported that he still became really anxious and that he didn’t know how to manage this (C4).

### Parents observed differences in anxiety

Parents described the changes in their children in relation to their anxiety and how they managed stress. They reported that their children were “more relaxed” (P10), and “a lot calmer” (P5, P8, and P10). A few parents specifically reported an increase in their child’s awareness; “It’s helped [her] realise her impact, like the way she behaves impacts everyone else in the area. It’s not just about how she feels” (P4), and another parent stated, “she’s definitely aware of when she is becoming anxious” (P3). One parent reported that their child was “stopping and thinking before reacting” (P4), describing the child’s improved ability to put space between feelings and actions. They also described an increase in flexibility; “Things tend to be really rigid, structured, and like there’s no flexibility for her. And then when we’d leave [the group], um, she’d just be a lot happier in general and relaxed” (P4). A few parents reported that the group had a positive impact on their child’s meltdowns, with one reporting “It’s easier to bring him down …the behaviour doesn’t last as long” (P2); another stated, “at its most intense it would have been up to an hour and a half two or three times a day… but we haven’t seen a tantrum of that length for a long time now” (P5); and another parent said, “little things like that would normally set him off, he’s just much better now” (P10). One parent described their child’s reduced anxiety, even when encountering regular causes of the anxiety; “It’s less, but the key triggers are still there” (P6).

Some parents reported that they did not notice a change in their children’s behaviour; “Unfortunately I haven’t seen that much difference” (P9). One parent reported that there were “not major changes, but just minor things” (P3). It was interesting that the parents’ reports of no changes in behaviours were not always consistent with what their child reported, and vice versa. A subgroup of the parents reported that there were other factors they believed contributed to the positive change in their children’s anxieties and behaviours. These included the child maturing, effects of medication, and changing schools. However, all parents reported that they believed mindfulness also played a part in the positive changes seen; as one parent said, “it’s all the small bits that make the whole” (P6).

Some parents described how the strategies used in the mindfulness group helped them to know how to respond differently to their child to better support them. One parent explained, “I found out in the group that some of the times it wasn’t *what* I was saying to him. It was *how* I was saying that to him. That’s sort of, I’ve gone in worked up but got him all anxious. It’s just learning to speak with a soft voice and obviously no yelling, no screaming, that’s just no good for anybody” (P10). One parent described that the mindfulness techniques even helped him feel less anxious “with the black dot on the hand, so when things get stressful or anxious, that definitely worked for me at work as well” (P10).

### Breathing and yoga helped

The techniques that most children found helpful were breathing and yoga. The children described how the techniques benefitted them, including that breathing “clears all of them [thoughts]” (C8) and that the yoga “made my body feel lighter” (C4). Most children reported that they enjoyed the yoga, breathing, and mindful eating. The children were divided on their perceptions of the other techniques; for example, some hated the mindful drawing activity whilst others loved it. Some parents recommended personalising the mindfulness techniques by making them relevant to each child’s unique strengths and interests.

Children and parents reported varying levels of success in applying the mindfulness techniques outside of the group. Some reported positive results “Having her use the drawing is really helpful. And the dragon breath” (P5). One parent described that his child did the breathing automatically without prompting when he felt upset; “He just takes himself off and does a little bit of deep breathing, breathing in and breathing out, and yeah, calms himself” (P10). Children described using the yoga and breathing techniques at home; “it distracts me from whatever I was thinking about” (C7). One parent reported that her child had started teaching her sibling the techniques; “[her sister] will be going crazy and she’ll say ‘Can you stop and just breathe for a minute? Breathe in, breathe out’” (P5). Some children had implemented some of the mindfulness in the classroom at school. One child described how he does sun salutations at school, and another said, “I do them [meditations] every day in school” (C5). Other children needed more prompting and support from their parents. One parent reported that his son “doesn’t really utilise them [the strategies] outside of class [the group]” (P6). Frequency of using the techniques varied among the children; one child described how she used the breathing techniques “almost all day” (C3), and a parent described that their child used “one [technique] at least every day at some point” (P2). Some children reported they could not remember the mindfulness techniques and so did not use them out of the group setting. The children’s descriptions of how much they used the techniques and/or how helpful they were was not always aligned with their parents’ perceptions.

### Parents wanted more

Parents reported they would have liked more support to encourage their children to generalise the learnings from the group into their home and community environments. Most parents felt they had a basic understanding of the mindfulness concepts used in the group intervention; however, reported they would have liked more support as they had limited prior knowledge of this intervention; “it’s our first time to join mindfulness… we actually have zero knowledge, but we love the idea” (P1). Parents described how they sometimes found it hard to know what concepts or strategies had been practiced in the group because their children often did not discuss with them directly what they had learnt; “he didn’t really tell us very much about it (laughing)” (P7).

Parents reported that they did not necessarily want more direct involvement in the group because they felt it was important for their children to have an opportunity to learn the strategies independently without parental oversight. In addition, some parents explained that having the time to themselves whilst their children were in the mindfulness group was important and meaningful for them: “I like to have a sit down, have a cup of coffee, and relax [laughter] and not worry about my child. It was good ‘cause it gave us a chance to chat [with other parents], relate and give each other advice” (P4). Another parent also reported they valued the time they had available while waiting as their child participated in the mindfulness group; “I get [sic] just as much out of the group as he did” (P2). However, parents would have liked more tools and strategies to remember the mindfulness techniques, as one parent said, “If I know the key words [used in the group] it probably would help” (P1).

Group facilitators ran 15 min debriefs for parents at the end of the group in the first week and sent home written summaries of the group content after each session. However, the parents had varied learning styles and whilst some described the debrief sessions as well run and helpful, others reported they would have preferred a booklet, emails, or easy to remember visuals or animated characters. One suggested that it would be helpful if the children “wrote down one thing that they liked, that made them feel better, that worked for them at the end of each session” (P9). Some parents advocated that it would be helpful to have more follow-up by the facilitators and strategies provided to support their child’s continued use of the mindfulness techniques after the group ended. As one parent disclosed, “I was given the documentation [written summaries], I came home and put into a file, and life went on” (P5).

### Challenges and benefits of group intervention

Having the mindfulness intervention in a group setting had overall benefits for the participants but also posed some challenges for them. A few parents described that it was sometimes hard to get their children to attend the group; “once he was there, he enjoyed it. But initially just getting him there was a bit of a mission” (P10). One child described that the lack of knowing what to expect from other participants meant attending the group could be anxiety-provoking for them, and another child described how the group made them feel “worried (C1).

A few children described they were “bored” in the group and instead wanted to engage in activities related to their interests (C4 and C6). From a social perspective, some children found it difficult to be in the group because they found the other group members “annoying” (C5). Many of the children reported that they did not like some aspects of the social environment the group setting created including loud noises, children not getting along or arguing with one another, and feeling like the room was too crowded. One child shared that he “doesn’t like being around lots of people” because he worries about them (C6). Some of the parents also agreed that they perceived there to be some negatives of the group setting, especially if their children showed signs of becoming distressed or not liking the other children.

Conversely, other participants described how they enjoyed the social side of the group-based intervention and that they used the opportunity to learn from the other children. Two children liked “meeting new people” (C1 and C2) and another described how the group provided him with a sense of community; “it helped me have a sense of… like…other people who… like… are also doing the same things” (C7). Parents and children alike acknowledged the value of the social aspect of the group-based mindfulness intervention; “It probably would have helped him a little, but not very much, if he had of been on his own” (P7), and “other kids challenged me and made me better” (C8).

## Discussion

This study investigated the feasibility of a mindfulness-based group intervention for children on the autism spectrum. The outcomes of anxiety and mindfulness attention showed improvements between pre-test and 2-month follow-up; however, these improvements were not statistically significant and with a small effect. The study may have been underpowered due to small sample who completed the outcomes measures (n = 10 children and n = 10 parents) to detect statistically significant changes over time. Individual item analysis indicated a significant increase in the children’s awareness of their emotions in the moment after the group as measured by the MAAS-A but this increase was not sustained at follow-up. As the parents suggested in the interviews, more support in implementing the strategies outside of the group setting may have supported this change to generalise and be maintained after the group finished. The findings from the qualitative data indicated that most children and the parents reported they perceived the children were calmer, had improved frustration tolerance, experienced fewer meltdowns, and had increased awareness of their emotions and their impact on others. However some did not experience any changes in their anxiety.

It is worth exploring what the ideal outcome measure is for a mindfulness intervention. A previous study that used the MAAS-A for evaluating the MYmind mindfulness program for children on the autism spectrum also did not find a significant difference at post-test (de Bruin et al., 2015). The authors hypothesised that this might have been because the MAAS-A measures trait mindfulness and that a better outcome measure may be state mindfulness because the short length of the study may not have allowed for mindfulness to become a trait. Another study on the MYmind program also found no significant difference in children’s reports of mindful awareness (Ridderinkhof et al., 2018). Both of these prior studies found significant short (2 month) and long-term (one year) differences in the children’s function, including social communication, internalising, externalising, attention (Ridderinkhof et al., 2018), and cognitive function and communication (de Bruin et al., 2015). Therefore, a measure of functional performance may be a better indication of the effectiveness of mindfulness interventions for children on the autism spectrum. This may have contributed to the null results of the study. This study found no significant difference in children’s anxiety (based on ASC-ASD and ASC-ASD-P scores) after completing the 10-week mindfulness program. An anxiety outcome measure was included in the study because the participants had a goal of reducing their anxiety. However interestingly, previous studies determining the effectiveness of mindfulness programs on children and adolescents on the autism spectrum have not utilised anxiety as an outcome. Other studies have used social communication, emotional and behavioural functioning, mindfulness and attention (Ridderinkhof et al., 2018; Ridderinkhof et al., 2020), quality of life, worry and rumination (de Bruin et al., 2015) and problem behaviours (Hwang et al., 2015). It would be worth exploring further whether anxiety is a suitable outcome measure in this population in future studies, as mindfulness intervention has been found to reduce anxiety in the general population (Hofmann et al., 2010).

The parents reported that they wanted more information about mindfulness and how to support the children to translate the strategies into their everyday lives. The Mindful Movers program described in this study did not train the parents in mindfulness. This differed to a previous mindfulness program for children on the autism spectrum that also trained the parents in a 9-week mindfulness program separately to their children’s program (de Bruin et al., 2015; Ridderinkhof et al., 2018). In another study, the mothers of children on the autism spectrum were trained in mindfulness techniques first and then the mothers provided ‘parent-mediated’ home-based mindfulness training to their children with support from clinicians (Hwang et al., 2015). These previous studies found significant functional improvements for the child. Additionally, teaching mindfulness to parents resulted in significant improvements in the parent’s quality of life, stress, and anxiety (de Bruin et al., 2015; Hwang et al., 2015; Ridderinkhof et al., 2018). These findings are consistent to those of other studies that found mindful parenting led to decreased stress and depression among parents of children on the autism spectrum (Beer et al., 2013). Therefore, when considering the parents in our study reported a desire for more information about the mindfulness program and the strength of existing literature, it appears that training parents in mindfulness should incorporated in future mindfulness programs for children on the autism spectrum. How this parent training is best achieved warrants further investigation. Finally, the parents in our study requested more specific strategies for embedding mindfulness into their child’s everyday life. This may be achieved through daily reminders on mobile devices, as a previous study found that sending participant’s daily reminders to complete mindfulness tasks resulted in a reduction in overall stress levels (Morrison et al., 2017).

The qualitative component of this study elicited information about how mindfulness programs may need to be modified to meet the specific needs of children on the autism spectrum. Overall, breathing and yoga seemed to work for most children in this group whereas other strategies were more divisive. Parents advised that tailoring the strategies to the child’s interests would further facilitate uptake of mindfulness. Whilst the group setting did pose challenges for some participants, others reported that they found interacting with peers to be a helpful aspect of the group, a finding which has been confirmed by other studies exploring mindfulness interventions for adolescents on the autism spectrum (Ridderinkhof et al., 2019). However it would be helpful to customise the environment and format of the group to accommodate the sensory and communication needs of the group members. For example, having a quiet space available if and when the children become overwhelmed, and providing resources prior to the commencement of the group on what to expect may be helpful (Fletcher-Watson & May, 2018).

### Limitations and recommendations for future research

This study had a small sample size, which may have produced a Type II error and the inability to statistically detect significant changes in the pre-post scale measures. More studies exploring the potential benefits of mindfulness interventions with children on the autism spectrum are needed and should include a larger sample size and a control group. In addition, it would be worth exploring the optimal duration for mindfulness groups, as the limited number of sessions of the current group may have had an impact on the outcomes. This study did not collect information about adaptive functioning and psychiatric comorbidities for the children who participated in the group. Future studies evaluating the effectiveness of mindfulness interventions for children on the autism spectrum should include this. Race and ethnicity of children and caregivers was not reported for this study. This may limit generalisability. The group included fourteen children who ranged from 10 to 14 years of age. Future research should explore the optimal number of participants per group, as well as well as considering the best division in terms of age. It is recommended future studies collect quantitative data about whether children used the mindfulness techniques outside of the group sessions, in the form of a self-reported rating scale. This would have provided further information about the extent that mindfulness skills were integrated into everyday life. This study did not train parents in mindfulness, and parents reported they wanted to learn how to support their children to generalise their mindfulness skills to outside of the group. Therefore, future mindfulness interventions should include a component that teaches the parents mindfulness. There is also a need for studies that explore how to effectively teach parents mindfulness alongside their children. The ideal outcome measures for mindfulness intervention for children on the autism spectrum needs to be explored further. Measures of functional performance may be more helpful than using measures of anxiety and mindfulness outcomes (Ridderinkhof et al., 2018). The existing evidence pertains to the use of mindfulness interventions with children aged 8 + years (de Bruin et al., 2015; Hwang et al., 2015; Ridderinkhof et al., 2018). Research into the feasibility, acceptability, and benefits of mindfulness for younger children on the autism spectrum and their families in warranted.

## Conclusions

This study explored the feasibility of a group-based mindfulness intervention with 14 children on the autism spectrum. The study found no significant differences in the participants’ scores on the quantitative measures of anxiety, mindful attention, and well-being before and after participating in the intervention, which may be attributed to a Type II error related to the small sample size. Qualitative data indicated that some of the children found the intervention helpful to reduce their anxiety and improve their tolerance to triggers that cause anxiety and frustration. Caregivers wanted more information about mindfulness techniques; consistent with the findings of previous research that teaching caregiver’s mindfulness alongside children is important for success of the intervention. Some children found the group setting challenging. Adaptations to make the group setting more supportive and personalisation of some mindfulness activities to align with the unique strengths and interests of children on the autism spectrum is a consideration for future practice and research. .

## Appendix 1

**Semi-structured Interview guide – Parents**.

1. Could you tell me about your experience as a parent of a child in the Mindful Movers program?

*Prompts*.

- *What did you think about the structure of the session?*
- *What did you think about the overall structure of the program?*
- *Did you think the location was appropriate? Would you suggest any changes to the environment?*
- *What did you think of the group setting?*

2. What did you perceive your child’s experience of the group to be?

*Prompts*.

- *What did you think he/she liked?*
- *What did you think he/she didn’t like?*

3. What are your thoughts on the level of involvement in the group for you as a parent?

*Prompts*.

- *Would you suggest any changes?*

4. Have you and your family experienced any benefits from the program?

*Prompts*.

- *Were there any differences between the group sessions?*
- *Were there any differences after the group program?*
- *What do you think of the techniques used and taught?*
- *Was there any impact at home in everyday life?*
- *How did you apply the techniques taught at home?*
- *How does it affect your whole family dynamic?*

5. Were there any aspects of the program that did not work for your child or your family?

*Prompts*.

- *Is there anything you didn’t like about the group?*
- *Were there any negative outcomes?*

6. What is your perspective of the impact of the program on your child’s anxiety levels?

*Prompts*.

- *What have you noticed as differences in your child’s anxiety levels?*
- *What have you observed your child doing differently since the group?*

7. Is there anything else you would like to say?

## Appendix 2

**Semi-structured Interview guide – Children**.

1. Can you tell me a bit about what you have been doing at the mindfulness group for the last 10 weeks? (note - photos of the group will be used for prompting)

*Prompts*.

- *What do you do in the MiMo group?*
- *Who was there at the MiMo group?*

2. What did you think of the group program?

*Prompts*.

- *What was your favourite thing to do at the group?*
- *What was your least favourite thing to do at the group?*
- *Did you like having people with you?*
- *Did you like the therapists who run the group?*
- *Did you feel comfortable talking in the group?*

3. What emotion did the group make you feel?

*Prompts*.

- *Why did it make you feel that?*

4. Have you noticed any differences in how you are feeling since the group? Can you tell me about this?

*Prompts*.

- *What have you used from the group at home?*

5. Is there anything else about the group you would like to tell me?
